# Anti-hyperglycemic and anti-hyperlipidemic effects of guava leaf extract

**DOI:** 10.1186/1743-7075-7-9

**Published:** 2010-02-02

**Authors:** Yoriko Deguchi, Kouji Miyazaki

**Affiliations:** 1 Yakult Central Institute for Microbiological Research, 1796 Yaho, Kunitachi-shi, Tokyo 186-8650, Japan

## Abstract

*Psidium guajava *Linn. (guava) is used not only as food but also as folk medicine in subtropical areas around the world because of its pharmacologic activities. In particular, the leaf extract of guava has traditionally been used for the treatment of diabetes in East Asia and other countries. Moreover, the anti-hyperglycemic activity of the extract has been reported in some animal models. However, little is known regarding the therapeutic activity of the extract in human clinical trials as well as its underlying therapeutic mechanisms and safety. In Japan, Guava Leaf Tea (Bansoureicha^®^, Yakult Honsha, Tokyo, Japan) containing the aqueous leaf extract from guava has been approved as one of the Foods for Specified Health Uses and is now commercially available. This review describes the active component of the aqueous guava leaf extract and its inhibition of alpha-glucosidase enzymes *in vitro*, safety of the extract and Guava Leaf Tea, reduction of postprandial blood glucose elevation, and improvement of hyperglycemia, hyperinsulinemia, hypoadiponectinemia, hypertriglycemia and hypercholesterolemia in murine models and several clinical trials. It is suggested that the chronic suppression of postprandial blood glucose elevation is important in preventing type 2 diabetes mellitus, and that Guava Leaf Tea is considered useful as an alimentotherapy for chronic treatment.

## Background

The number of diabetes mellitus (DM) cases has been increasing worldwide in recent years. In 2000, the World Health Organization estimated a total of 171 million (2.8%) people with DM from the global population, and this figure has been projected to increase to 366 million (4.4%) by 2030 [1]. In particular, cases of type 2 DM (T2DM) have been increasing in contrast to cases of type 1 DM (T1DM), an autoimmune disease resulting in the destruction of insulin-producing beta cells of the pancreas and the failure to produce insulin. In more developed countries, the cure and prevention of T2DM have become important concerns. On the other hand, T2DM is expected to become a more serious problem in developing countries because of the trend of urbanization and consequent lifestyle changes, perhaps most importantly exemplified by a shift to the "Western-style" diet, which is high in fat.

T2DM is generally characterized by hyperglycemia, insulin resistance (reduced insulin sensitivity) and obesity. Obesity is associated with not only T2DM but also hyperlipidemia and hypertension. Coexistence of these diseases is well known as metabolic syndrome, a high risk factor for cardiovascular disease [2-4]. Insulin resistance is considered a key feature of these diseases and is defined as a state requiring more insulin in order to obtain the biological effects achieved with a lower insulin level in the normal state. This metabolic abnormality is induced by obesity, especially increased visceral fat, via the enhancement of inflammation and hypoadiponectinemia. Adiponectin is an adipocytokine specifically and abundantly expressed in adipose tissue which directly sensitizes to insulin, and its level is inversely correlated to the percentage of body fat in adults. Treatment with thiazolidine derivatives, one type of anti-diabetic drugs, ameliorates insulin resistance and increases serum adiponectin level in T2DM patients with hypoadiponectinemia [5]. Therefore, improvement of insulin resistance and hypoadiponectinemia is expected to be an effective therapeutic strategy for the improvement and/or prevention of T2DM as well as metabolic syndrome.

The common guava tree (*Psidium guajava *Linn.) is a member of the Myrtaceae family, which is native to tropical and subtropical countries. Its fruit is commonly used as food and processed as juice and jam. The other common use of *Psidium guajava *Linn. (guava) is as folk medicine. Aside from these uses, Gutiérrez et al. [6] have reviewed the potential pharmacologic activities of the extract from the fruit, leaf, bark or roots; these activities include antioxidant, hepatoprotective, anti-allergy, anti-microbial, anti-genotoxic, anti-plasmodial, cytotoxic, anti-spasmodic, cardioactive, anti-cough, anti-diabetic, anti-inflammatory and anti-nociceptive activities in vitro and/or in animal models. Interestingly, guava leaves have also attracted attention as a folk remedy for diabetes not only in Japan and East Asia [7-9] but also in Africa [10]. However, little is known regarding the anti-hyperglycemic or anti-diabetic activity of the guava leaf extract in clinical trials, except for traditional uses [8] and the effects of a single ingestion of guava juice in healthy volunteers and a few diabetic subjects [11]. Moreover, its underlying therapeutic mechanisms and safety in terms of interaction with other medicines remain unclarified.

For the maintenance and promotion of health and the prevention of lifestyle associated disease, the Japanese Ministry of Health, Labor and Welfare first published "Foods for Specified Health Uses" (FOSHU). FOSHU lists foods whose claims of their physiological effects on the human body have been officially approved and such foods were legally permitted to be used as dietary products for health preservation [12]. Guava Leaf Tea (Bansoureicha^®^, Yakult Honsha, Tokyo, Japan), which contains the aqueous guava leaf extract (GvEx), has been approved as FOSHU and recommended for subjects with pre-diabetes; it is presently commercially available in Japan [13].

This article reviews evidence regarding the anti-hyperglycemic activities and safety of GvEx and Guava Leaf Tea in vitro, as well as in animal models and several clinical trials. It also describes the efficacy and safety of Guava Leaf Tea in pre-diabetic and diabetic patients with T2DM.

## Review

### 1. Inhibition of alpha-glucosidase enzymes by GvEx and its active component

In the digestive tract, some alpha-glucosidase enzymes, such as alpha-amylase, maltase and sucrase, digest carbohydrates to glucose that can be absorbed through the intestine. Alpha-glucosidase inhibitors (alpha-GIs), namely, acarbose and voglibose, prevent the digestion of carbohydrates based on competitive enzyme inhibition, and provide short-term glycemic control [14].

**Figure 1:**
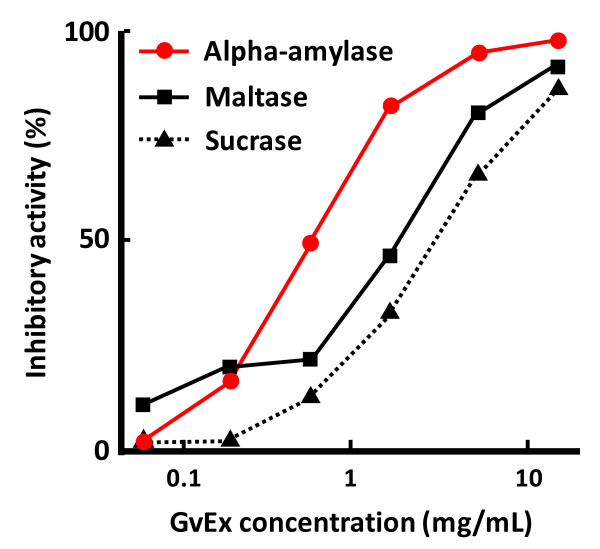
**Dose-dependent inhibition of alpha-amylase, maltase and sucrase by GvEx**. Enzyme reaction was conducted for the specified time, and the amount of glucose or maltose produced was measured by HPLC. Maltase and sucrase derived from acetone powder of rat intestine, and alpha-amylase obtained from porcine pancreas were purchased from Sigma Chemical Co. (St. Louis, MO, USA) [15].

Deguchi et al. [15] demonstrated that GvEx, which was prepared by hot water extraction from guava leaves, inhibited the in vitro activities of maltase, sucrase, and alpha-amylase in a dose-dependent manner (Fig. 1). The 50% inhibitory concentration (IC50) of GvEx was 0.6 mg/mL for alpha-amylase, 2.1 mg/mL for maltase, and 3.6 mg/mL for sucrase, indicating the higher inhibitory activity of alpha-amylase than the other two enzymes. Furthermore, Wang et al. [16] found that the aqueous extract from guava leaves inhibited both sucrase and maltase activities in the small intestinal mucosa of diabetic mice, occurring as a mixed type of competitive and non-competitive inhibition.

In order to clarify the active component of the guava leaf extract, GvEx solution was fractionated in dialysis tubes of 5,000 and 30,000 MW pore size. The inhibitory activity of alpha-amylase was detected in the fraction with a MW between 5,000 and 30,000. This fraction reacted with ferrous tartrate, indicating that a component with a phenolic hydroxyl group was part of the fraction [17]. It has been reported that guava leaf contains some polyphenols, such as peduncladgin, casuarinin and isostrictinin [18-20]. However, high-performance liquid chromatography (HPLC) analysis demonstrated that these elementary polyphenols were present in the ethyl acetate extract of guava leaf but not in GvEx. Several instrumental analyses, such as ^1^H-nuclear magnetic resonance (NMR), infrared absorption spectrum, and solid ^13^C-NMR, have suggested that the active component of GvEx was a polymerized polyphenol named guava leaf polyphenol (GvPP), which is composed of ellagic acid, cyanidin and other low-molecular-weight polyphenols [17].

Here, it was shown that the GvEx acts as an alpha-GI in vitro and contains GvPP as the active component.

### 2. Reduction of postprandial blood glucose elevation

#### 2.1 Murine models

It is well known that orally administered alpha-GIs have the potential to reduce postprandial blood glucose elevation as shown in a carbohydrate loading test in vivo [21-24]. To determine the effect of GvEx on postprandial blood glucose elevation, normal mice were immediately given GvEx or saline (control) following overnight fasting. Thirty minutes later, soluble starch, sucrose or maltose (2 g/kg) was loaded and blood glucose level was measured at 30-min intervals from 0 to 120 min. Compared with control, the single ingestion of GvEx significantly reduced the area under the curve (AUC) of postprandial blood glucose levels by 37.8% after loading soluble starch at 250 mg/kg and by 31.0% and 29.6% after loading sucrose and maltose, respectively at 500 mg/kg each [15]. Additionally, in streptozotocin-induced diabetic mice with a fasting blood glucose (FBG) level of >200 mg/dL, which is a model of T1DM, the single ingestion of GvEx (250 mg/kg) significantly reduced the AUC after loading soluble starch (unpublished data). Furthermore, anti-hyperglycemic activity was detected in animal models with T1DM [10,25,26].

#### 2.2 Human trials with normal, pre-diabetic and type 2 diabetic subjects

**Figure 2:**
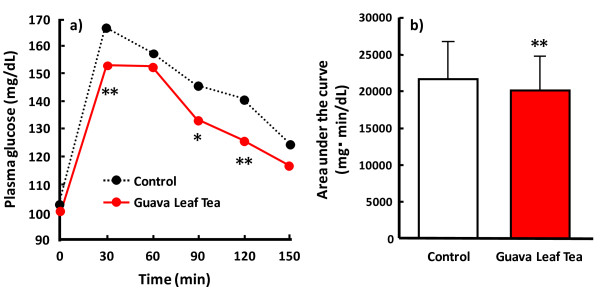
**Effect of single ingestion of Guava Leaf Tea on postprandial blood glucose elevation in human subjects**. (a) Time course of changes in blood glucose level. (b) Area under the curve in changes in blood glucose level. Nineteen subjects aged over 40 years, with or without pre-diabetes, with an FBG level of 103.0 ± 14.3 mg/dL and a body mass index (BMI) of >22.0 were recruited. After overnight fasting for 11 hours, the subjects ingested 200 g of cooked rice as a loading carbohydrate together with a bottle (190 mL) of hot water at week 1 and then with the same volume of Guava Leaf Tea containing about 400 mg of GvEx at week 2. Blood glucose level was measured at 30-min intervals for up to 150 min after ingestion. *: p < 0.05, **: p < 0.01 (paired *t*-test) [15].

According to the preparation method of GvEx, Guava Leaf Tea was manufactured for human trial. To clarify the above-mentioned findings in animal models, a crossover study was designed to evaluate the effects of a single ingestion of Guava Leaf Tea on postprandial blood glucose elevation in normal and pre-diabetic subjects [15]. The single ingestion of Guava Leaf Tea significantly reduced postprandial blood glucose elevation at 30, 90 and 120 min (Fig. 2a). In addition, the AUC of the glucose level after carbohydrate loading (ingestion of cooked rice) was significantly reduced by about 20% compared with control (Fig. 2b).

**Figure 3:**
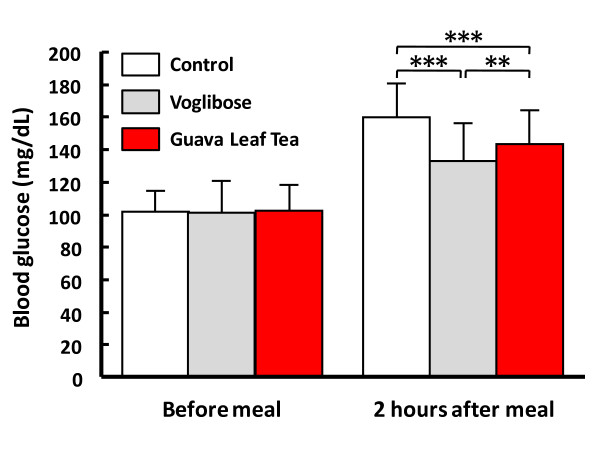
**Effects of Guava Leaf Tea and voglibose on postprandial blood glucose elevation in patients with T2DM**. Three days before and during the trial period, all patients with T2DM were treated with their usual anti-diabetic medication, namely, insulin, sulfonylurea and/or biguanide except for alpha-GI, followed by dietary treatment and/or exercise to control the FBG level at approximately 100 mg/dL. The subjects were randomized to group A or B and received their usual treatment only on day 1 (control). On day 2, the subjects in groups A and B were administered Guava Leaf Tea (200 ml) with meal and voglibose (3 mg) before meal, respectively. On day 3, the subjects were crossed-over for the administration of other treatment. The FBG and postprandial blood glucose levels were assayed before and 2 hours after meal, respectively. The combined data was analyzed using the Tukey test. **: p < 0.01, ***: p < 0.001 [27].

A crossover clinical trial involving 20 hospitalized patients with T2DM was conducted to compare the potential of Guava Leaf Tea and voglibose (Basen^®^; Takeda Chemical Industries, Ltd., Tokyo, Japan) to reduce postprandial blood glucose elevation [27]. As shown in Figure 3, the postprandial glucose level 2 hours after meal was elevated to ca. 160 mg/dL in the patients with each standard treatment (in control). The elevated level was significantly reduced with the single administration of Guava Leaf Tea and voglibose to 143 mg/dL (p < 0.001) and 133 mg/dL (p < 0.001), respectively. The reducing potential was significantly milder with Guava Leaf Tea than with voglibose (p < 0.01). There were no side effects, such as hypoglycaemia, due to abnormal interaction in the combined administration of each standard treatment and voglibose or Guava Leaf Tea [27].

Taken together, these findings, suggest that the single ingestion of GvEx or Guava Leaf Tea can reduce postprandial glucose elevation via the inhibition of alpha-glucosidase in mice and human subjects with or without diabetes. Furthermore, Guava Leaf Tea was found to have milder activity than voglibose.

### 3. Improvement of diabetes symptoms and hyperlipidemia

#### 3.1 Diabetic animal models

In previous studies, patients with T2DM were treated with alpha-GIs, namely, acarbose [23] or voglibose [24] because long-term oral administration has been reported to improve diabetic markers [e.g., hemoglobin A_1c _(HbA_1c_) and insulin resistance]. Deguchi et al. [15] previously examined the effects of consecutive ingestion of GvEx for 7 weeks on the progression of T2DM and nephropathy in genetically diabetic mouse models (i.e., C57BL/Ksj, db/db, and *Lepr*^*db*^/*Lepr*^*db*^). They showed that the mice developed widespread pathological abnormalities including not only diabetes and obesity but also well-defined nephropathy [28,29]. Compared with drinking water (control), the GvEx (250 mg/kg/day) solution significantly reduced blood HbA_1c_% after ingestion for 5 and 7 weeks. GvEx also improved nephropathy with a significant reduction in the thickening index of the glomerular mesangial matrix in the kidney observed at 7 weeks (Table 1). In contrast, there were no significant effects on weight gain, food and water intakes of the diabetic mice.

**Table 1:** Effect of GvEx on glomeruli in db/db mice

	Glomeruli
	
Group	Number of thickened matrix ^1)^
Control (n = 10)	11.3 ± 2.7
GvEx (n = 9)	7.2 ± 2.6 **

Six-week-old db/db (C57BL/KsJ) mice were equally divided into 2 groups according to body weight. All animals were permitted water or GvEx solution (250 mg/kg bw/day) mixed in tap water and diet (MF commercial diet, Oriental Yeast Co., Ltd., Tokyo, Japan) ad libitum. The study period was 7 weeks [15].

^1) ^Number of glomeruli per specimen with moderate and marked changes.

Values are expressed as mean ± SD. **: p < 0.01 (Student's t-test).

Recently, Shen et al.[30] have reported the effects of the aqueous extract from guava leaves on type 2 diabetic rats. They showed that long-term feeding of the extract significantly reduced blood glucose level, increased plasma insulin level in an oral glucose tolerance test, and stimulated activities of some glucose metabolic enzymes. Additionally, the single feeding of the extract significantly reduced blood glucose level in an oral glucose tolerance test. However, the underlying mechanisms have not yet been clearly elucidated.

The above-mentioned findings indicate that the consecutive ingestion of GvEx has the potential to improve diabetes symptoms such as hyperglycemia, nephropathy and insulin resistance in diabetic animal models.

#### 3.2 Human trials in pre-diabetic and type 2 diabetic patients

To confirm previous findings in diabetic animal models, a first long-term clinical trial was conducted to evaluate the effects of consecutive ingestion of Guava Leaf Tea with every meal for 12 weeks on the parameters of diabetes symptoms and safety in 15 male subjects with pre-diabetes and mild type 2 diabetes [31].

Table 2 shows the serum parameters of diabetes and analysis results between the initial week and week 12 of ingestion of Guava Leaf Tea in subjects with pre-diabetes and mild diabetes. In all the subjects, the FBG level showed a decrease from 136 to 131 mg/dL (p = 0.07) and a significant reduction was detected after the calculation of reduction rate of the FBG level. In particular, the FBG level showed a more pronounced decrease in the pre-diabetic subjects (p = 0.06; n = 7; initial FBG level: 110-126 mg/dL). Five of seven pre-diabetic subjects showed a reduction in blood HbA_1c_%. Also, the levels of insulin, C-peptide and homeostasis model assessment for insulin resistance (HOMA-IR) significantly decreased in all subjects. Moreover, after the ingestion of Guava Leaf Tea for 12 weeks, serum levels of total cholesterol (T-CHO) and triglyceride (TG) significantly decreased in the subjects with hypercholesterolemia and hypertriglycemia (n = 7 and n = 5; initial levels: >220 mg/dL and >150 mg/dL, respectively). There were no abnormal changes in the parameters of iron metabolism, liver and kidney functions, blood chemistry and on items covered in the physician's physical examination and health interviews during this trial [31].

**Table 2:** Effect of consecutive ingestion of Guava Leaf Tea on serum parameters of carbohydrate metabolism in pre- and mild diabetic patients and diabetic patients with T2DM under anti-diabetic medication

Serum parameter	Pre- and mild diabetic patients ^1)^			Type 2 diabetic patients ^2)^		
		
	n	Initial week	Week 12	n^3)^	Initial week	Week 8
FBG (mg/dL)	15	136 ± 22	131 ± 25 ^#1^	21	183 ± 71	171 ± 76
111-127 mg/dL	7	118 ± 7	112 ± 6 ^#2^			
Reduction rate of FBG (%)	15	0	4.3 ± 7.6*			
HbA_1c_(%)	15	6.1 ± 0.7	6.2 ± 0.8	22	7.4 ± 1.4	7.4 ± 1.3
≧ 6.5%				15	8.1 ± 1.1	7.8 ± 1.1*
Insulin (μU/mL)	15	9.0 ± 3.0	7.0 ± 2.0**	19	28.7 ± 26.3	21.5 ± 18.0
≧ 17 μU/mL				11	43.6 ± 25.5	27.5 ± 19.4*
HOMA-IR	15	3.1 ± 1.3	2.3 ± 1.0**	19	12.2 ± 9.9	10.8 ± 13.0

^1) ^Guava Leaf Tea (190 ml/bottle) was ingested 3 times/day with each meal for 12 weeks by pre-diabetic and mild type 2 diabetic subjects who were 15 males, over 45 years of age and who had an FBG level of 110 mg/dL or higher and a BMI of >22.0.

^2) ^Guava Leaf Tea (200 ml/bottle) was ingested 3 times/day with every meal for 8 weeks by 22 patients with T2DM who had a blood HbA_1c_% of >6.0% before intake. Of these 22 patients, 19 received anti-diabetic medication (insulin, sulfonylurea and/or alpha-GI) throughout the trial. Three patients with hypercholesterolemia were also administered fluvastatin, an inhibitor of HMG-CoA reductase in addition to an anti-diabetic drug throughout the trial.

^3) ^Some data under the no fasting condition are omitted.

Values are expressed as mean ± SD. *: p < 0.05, **: p < 0.01, ^#1^: p = 0.07, ^#2^: p = 0.06 (Dunnett's multiple paired test). Reproduced from [15,32] with some modifications.

A second long-term clinical trial investigated the effects of consecutive ingestion of Guava Leaf Tea for 8 weeks on the parameters of diabetes symptoms and safety in diabetic patients receiving therapy, that is, anti-diabetic medication with or without an inhibitor of HMG-CoA reductase [32].

Table 2 shows the serum parameters of diabetes and analysis results between the initial week and week 8 of ingestion of Guava Leaf Tea in diabetic patients with some medication. Ingestion of the tea significantly decreased blood HbA_1c_% in diabetic patients who had initial values of >6.5% and were assessed to have abnormal control of blood glucose level. Additionally, the ingestion of the tea significantly reduced serum insulin level in diabetic patients with hyperinsulinemia whose serum insulin level was >17 μU/ml before intake. The ingestion of the tea also decreased the parameter values of lipid metabolism, that is, triglycerides (TG; p < 0.05, for 4 weeks), nonesterified fatty acids (NEFAs; p < 0.05, for 4 weeks), remnant-like particle-cholesterol (p = 0.08, for 4 weeks) and phospholipids (p = 0.06, for 8 weeks), in the subjects with values higher than the reference values in patients without fluvastatin treatment. In contrast, neither side effects resulting from alterations in the parameter values of liver and kidney functions or blood chemistry nor changes in doctor's health interviews were observed during the entire clinical trial period. Also, there was no hypoglycemia due to the abnormal interaction between Guava Leaf Tea and anti-diabetic drugs with or without an HMG-CoA reductase inhibitor [32].

Taken together, it is suggested that the consecutive ingestion of Guava Leaf Tea with every meal improves diabetes symptoms, such as hyperglycemia, hyperinsulinemia, insulin resistance as well as hyperlipidemia in pre-diabetic and mild diabetic patients with or without hyperlipidemia. Moreover, it is indicated that the consecutive ingestion of Guava Leaf Tea in addition to anti-diabetic and anti-hypercholesterolemia medications shows no side effects due to the abnormal interaction.

### 4. Improvement of hypercholesterolemia and hypoadiponectinemia

To verify the anti-hyperlipidemic activity of Guava Leaf Tea, a third long-term clinical trial investigated the effects of consecutive intake for 8 weeks on the parameters of hyperlipidemia, diabetes and safety in 23 subjects with borderline or mild hyperlipidemia with or without T2DM. During the trial, 7 subjects were administered fluvastatin, pravastatin, pitavastatin, colestimide (an inhibitor of cholesterol absorption) or ethyl icosapentate (a TG reducer) [33].

Table 3 shows the changes in serum lipid parameters after the ingestion of Guava Leaf Tea for 8 weeks in subjects with hypercholesterolemia (initial T-CHO level: >220 mg/dL). The consecutive ingestion reduced the serum levels of T-CHO (p < 0.05), LDL-cholesterol (LDL-CHO) (p = 0.06) and phospholipid (p < 0.05) in these subjects. A significant reduction in T-CHO level (p < 0.05) was also observed in the same subjects receiving no medicinal treatment. On the other hand, the levels of high-density lipoprotein cholesterol (HDL-CHO), TG, NEFA and lipid peroxide were not significantly changed in the same subjects. In contrast, the consecutive ingestion decreased the serum level of TG (p < 0.05, week 4) in subjects with hypertriglycemia (initial TG level: >150 mg/dL) and that of phospholipid (p < 0.05, weeks 4 and 8) in subjects with hyperphospholipidemia (initial phospholipid level: >250 mg/dL).

**Table 3:** Effect of consecutive ingestion of Guava Leaf Tea on serum lipid parameters in subjects with hypercholesterolemia

Serum parameter	n	Initial week	Week 8
T-CHO (mg/dL)	16	249 ± 20	235 ± 30q *
Without medical treatment	10	248 ± 21	231 ± 28*
LDL-CHO (mg/dL)	16	155 ± 23	143 ± 31 ^#^
HDL-CHO (mg/dL)	16	54 ± 13	52 ± 13
TG (mg/dL)	16	212 ± 113	202 ± 105
NEFA (mEq/L)	14	0.62 ± 0.26	0.47 ± 0.25
Phospholipid (mg/dL)	14	263 ± 26	246 ± 19 *
Lipid peroxide (nmol/mL)	14	0.5 ± 0.4	0.4 ± 0.2

Twenty-three subjects with moderate hyperlipidemia (T-CHO level of >180 mg/dL or TG level of >150 mg/dL) before intake ingested Guava Leaf Tea (200 mL) with every meal for 8 weeks. Data were stratified by an initial serum T-CHO level of >220 mg/dL. Values are expressed as mean ± SD. *: p < 0.05, #: p = 0.06 (Dunnett's multiple paired test). Reproduced from [33] with some modifications.

Moreover, the ingestion of Guava Leaf Tea significantly reduced blood HbA_1c _% in diabetic subjects (initial HbA_1c_%: >6.5%), and significantly increased serum adiponectin level in each subject with hypoadiponectinemia (Table 4) and hyperglycemia. The nutritional intake of all the subjects showed no significant variation in the results of the questionnaires that were designed for 1 week in the first, middle and last weeks of the trial period [33]. This suggests that the trial findings were due to the effects of ingestion of Guava Leaf Tea and not from nutritional intake. There were no abnormal changes in the parameters of liver and kidney function, blood chemistry and doctor's health interviews during the entire trial period. Also, side effects such as hypoglycemia due to the abnormal interaction between Guava Leaf Tea and an HMG-CoA reductase inhibitor, colestimide (an inhibitor of cholesterol absorption) or ethyl icosapentate [33] were not observed.

**Table 4:** Effect of consecutive ingestion of Guava Leaf Tea on serum adiponectin level in subjects with moderate hyperlipidemia (stratified by the initial level of serum adiponectin and HbA_1c_)

	Serum adiponectin level (μg/mL)		
	
	n	Initial week	Week 8
Initial adiponectin level			
< 4.0 μg/mL	5	2.3 ± 1.7	5.7 ± 1.8*
4.0 -- 5.5 μg/mL	3	4.5 ± 0.7	4.9 ± 3.9
5.5 -- 7.0 μg/mL	6	6.4 ± 0.4	6.8 ± 2.4
≧ 7.0 μg/mL	9	11.6 ± 4.8	13.0 ± 6.0
			
Initial HbA_1c_			
≧ 6.5%	9	5.7 ± 1.8	6.8 ± 3.2*
< 6.5%	14	10.4 ± 5.3	5.7 ± 1.8

Subjects and conditions in the intervention are described in Table 3. Values are expressed as mean ± SD. *: p < 0.05 (Dunnett's multiple paired test). Reproduced from [33] with some modifications.

Overall, the results indicate that the consecutive ingestion of Guava Leaf Tea together with every meal improves not only hyperglycemia but also hypoadiponectinemia, hypercholesterolemia and hyperlipidemia in pre-diabetic and diabetic patients with or without hyperlipidemia. The consecutive ingestion also ameliorates high blood cholesterol level in subjects with hypercholesterolemia or borderline hypercholesterolemia.

### 5. Safety

To confirm the safety of GvEx and Guava Leaf Tea, several toxicity studies have been conducted in vitro, as well as in animal models and human subjects.

In single-dose and 1-month repeated dose toxicity studies, **Kobayashi et al**. [34] demonstrated that the oral administration of GvEx (200 and 2000 mg/kg/day) caused no abnormal effects in rats, indicating that there is neither acute nor chronic toxicity. **Oyama et al**. [35] investigated the mutagenic activity of both GvEx and Guava Leaf Tea. They found that Guava Leaf Tea had a lower mutagenic activity than commercial green tea and black tea in a DNA repair test (Rec-assay); however, these teas showed no mutagenic activity in a bacterial reverse mutation test (Ames test). Moreover, GvEx did not induce chromosomal aberrations in a micronuclear test using peripheral blood erythrocytes, which were prepared from mice by a single oral administration of GvEx (2000 mg/kg). From these findings, it is suggested that Guava Leaf Tea and these commercial teas have no genotoxicity.

After the approval as FOSHU, Guava Leaf Tea has been taken by not only subjects with pre-diabetes but also patients with mild and moderate T2DM. If the ingested Guava Leaf Tea interacts with anti-diabetic and other commercial drugs, it poses a risk of side effects, such as hypoglycemia or inhibition of drug activity. Cytochrome P450 (CYP) isoforms in the human liver or the small intestinal epithelium are known to be involved in drug metabolism. Especially, CYP2C8, CYP2C9 and CYP3A4, which are typical CYP isoforms, can metabolize many commercial drugs. Grapefruit juice inhibits CYP isoforms and shows abnormal interaction with anti-diabetic drugs as well as many other commercial drugs, such as repaglinide and tolbutamide (sulfonylurea) as a stimulator of insulin release from the pancreas, sibutramine as an appetite suppressant, HMG-CoA reductase inhibitors, losartan as an angiotensin II receptor antagonist, calcium channel blockers, and warfarin [36,37]. Therefore, to evaluate the interaction between Guava Leaf Tea and anti-diabetic and other commercial drugs, **Kaneko et al**. [38] investigated the inhibitory effects of GvEx and Guava Leaf Tea on CYP2C8, CYP2C9 and CYP3A4 in vitro and compared them with quercetin as another inhibitor and grapefruit juice. Quercetin and grapefruit juice were shown to have higher inhibitory effects on CYP2C8, CYP2C9 and CYP3A4 than GvEx (more than 10-fold) and Guava Leaf Tea (more than 2- to 10-fold), respectively. A subsequent histopathological study showed the absence of response to the induction of P450 isoforms in the liver of rats with 1-month repeated oral administration of GvEx (2000 mg/kg/day). From these findings, it would appear unlikely that Guava Leaf Tea can cause drug interactions based on either inhibition or induction of cytochrome P450 isoforms.

**Deguchi et al**. [39] investigated the effects of GvEx in combination with typical alpha-GIs acarbose or voglibose on alpha-amylase activity in vitro and postprandial blood glucose elevation in mice. GvEx inhibited alpha-amylase dose-dependently when combined with the low active dose of acarbose or voglibose. When concomitantly administered with acarbose or voglibose to normal mice, acarbose and voglibose each at the active dose suppressed postprandial blood glucose elevation following loading of sugars with no effect of GvEx (250 mg/kg). In contrast, at the inactive dose, acarbose and voglibose did not affect the activity of GvEx (250 mg/kg). Therefore, these findings indicate that the combined ingestion of GvEx and an alpha-GI does not induce hypoglycemia in an animal model.

To further examine the effects of drinking excessive amounts of Guava Leaf Tea, human healthy subjects in a previous study [31] ingested a 3-fold volume (600 ml) of the tea. Notably, neither diarrhea nor hypoglycemia was observed. Furthermore, single ingestion and the consecutive ingestion of Guava Leaf Tea for 8 or 12 weeks with or without anti-diabetic and anti-hyperlipidemia drugs in human clinical trials demonstrated no side effects or abnormal changes, as described earlier.

These findings indicate that Guava Leaf Tea and GvEx induce neither toxicity, mutagenicity, nor abnormal interaction with anti-diabetic and anti-hyperlipidemia drugs, and have a lower potential for drug interactions based on either inhibition or induction of cytochrome P450 isoforms. Thus, Guava Leaf Tea and GvEx can be deemed food and a safe food material, respectively.

## 6. Discussion

Acarbose [21,23] and voglibose [22,24] are each administered as an oral hypoglycemic agent to improve postprandial hyperglycemia, fasting hyperglycemia and hyperinsulinemia. In a previous non-insulin-dependent diabetes mellitus trial (an international, multicenter, double-blind, placebo-controlled trial) in which about 1,400 pre-diabetic subjects with impaired glucose tolerance were enrolled [40,41], it was demonstrated that long-term treatment with acarbose decreases the risk of T2DM, hypertension and cardiovascular disease. Also, a recent randomized double-blind trial in Japan has proven that long-term treatment with voglibose reduces the risk of progression to T2DM in about 1,800 pre-diabetic subjects with impaired glucose tolerance [42]. It is important to point out that the development of T2DM is associated with the onset of cardiovascular disease. Currently, there is ongoing debate as to whether postprandial blood glucose level is a stronger predictor of cardiovascular events than fasting blood glucose in T2DM [43,44]. Therefore, the adequate control of postprandial blood glucose level is highly important in preventing the onset of T2DM or its complications in pre-diabetic or diabetic patients. To prevent the development of T2DM, pre-diabetic and non-diabetic subjects are firstly managed by modification of lifestyle in order to reduce body weight, without generally receiving pharmacologic drug therapy which is considered for higher-risk patients [45]. In this regard, both therapeutic exercise and alimentotherapy are attractive alternatives and recommended in both developed and developing countries. In Japan, FOSHU is particularly expected to aid in the prevention of T2DM.

On further evaluations in animal models and a human trial using subjects with or without pre-diabetes, a reduction in postprandial blood glucose elevation has been observed with a single oral ingestion of GvEx and Guava Leaf Tea [15] (Figure 2). In addition, the improvement of diabetes symptoms, such as hyperglycemia, nephropathy (Table 1) and insulin resistance, has been reported with the consecutive ingestion of GvEx in diabetic animal models [15]. Moreover, some clinical trials have demonstrated that the consecutive ingestion of Guava Leaf Tea with every meal improved diabetes symptoms and insulin resistance in pre-diabetic and diabetic patients [15,32] (Table 2). These observations indicate that the chronic suppression of postprandial blood glucose elevation, which is induced by the consecutive ingestion of Guava Leaf Tea containing GvEx via its alpha-glucosidase inhibition, plays an important role in the improvement of not only hyperglycemia and diabetes symptoms, as similarly induced by typical alpha-GIs, but also insulin resistance in pre-diabetic and diabetic patients.

A human study found that the consecutive ingestion of Guava Leaf Tea with every meal improved hypoadiponectinemia and hyperglycemia, showing an increase in adiponectin level and a decrease in HbA_1c_% in blood at each initial level [33] (Table 4). Recently, **Ochiai et al**. [46] have demonstrated that treatment of T2DM patients with acarbose for 12 weeks improves both hypoadiponectinemia and hyperglycemia, showing an increase in serum adiponectin level and a decrease in HbA_1c_% in blood. These findings suggest that Guava Leaf Tea has similar therapeutic potential to acarbose for improving hypoadiponectinemia and hyperglycemia. Moreover, there is a good inverse correlation between adiponectin and HbA_1c_% in blood of the patients treated with Guava Leaf Tea or acarbose. Although the exact mechanism remains uncertain, the improvement of hypoadiponectinemia and hyperglycemia is considered to be associated with the chronic suppression of postprandial blood glucose elevation by alpha-glucosidase inhibition.

A previous clinical trial has also demonstrated that the consecutive ingestion of Guava Leaf Tea together with every meal improved hypertriglyceridemia and hypercholesterolemia [33] (Table 3). It is speculated that the chronic suppression of postprandial blood glucose elevation is one of the underlying mechanisms involved in the improvement of not only hyperglycemia and hypoadiponectinemia but also hypertriglyceridemia and hypercholesterolemia. Hyperglycemia, hyperlipidemia and hypercholesterolemia are some of the criteria indicating metabolic syndrome and these conditions are closely associated with insulin resistance. It has been hypothesized that an increase in adiponectin level in hypoadiponectinemia improves insulin resistance, T2DM and metabolic syndrome [5]. Taking the results of all the clinical trials together, the consecutive ingestion of Guava Leaf Tea improves insulin resistance, hyperlipidemia and hypercholesterolemia with or without an increase in adiponectin level and a decrease in HbA_1c_%. Such consecutive ingestion may be expected to improve or prevent insulin resistance as well as metabolic syndrome. To confirm efficacy and safety, further large-scale clinical trials employing a larger number of subjects with metabolic syndrome are warranted. In particular, the enrolment of patients who received anti-hypertensive drugs and other commercial drugs is important to obtain more information on the absence of interaction between Guava Leaf Tea and these drugs.

In a crossover clinical trial of diabetic patients, it was demonstrated that Guava Leaf Tea has a milder reducing activity for postprandial glucose elevation than voglibose, a typical alpha GI. This is based on the lower activity of GvEx on sugar-degrading enzymes than voglibose. However, alpha-GIs have been reported to have side effects, such as diarrhea, meteorism, flatulence and abdominal wind [14,23,47]. One mechanism suggested to underlie these side effects is that the potent inhibition of sugar-degrading enzymes allows undigested sugars to reach the colon where they stimulate the abnormal production of several acids by intestinal bacteria. This is followed by a decrease in intestinal pH and the generation of carbon dioxide gas, which are responsible for the side effects. In contrast and as described earlier, no abdominal symptoms were observed after the consecutive ingestion of Guava Leaf Tea for 8 or 12 weeks in some human trials. In addition, previous in vitro studies and investigations using animal models have demonstrated that Guava Leaf Tea and GvEx induce neither toxicity nor mutagenicity. Moreover, both GvEx and Guava Leaf Tea have weak inhibitory activity on cytochrome P450 isoforms, which are associated with the metabolism of anti-diabetic drugs and many commercial drugs. This suggests that Guava Leaf Tea has a lower potential for abnormal interaction with these drugs. It is therefore suggested that Guava Leaf Tea containing GvEx is a useful and harmless food for treating pre-diabetic and diabetic patients.

## Conclusions

On the basis of numerous lines of scientific evidence regarding the effectiveness and safety of Guava Leaf Tea containing GvEx for treating T2DM, it was approved as FOSHU in March 2000 and it is recommended for individuals who are anxious about their high blood glucose and control of sugar uptake. Many Japanese consumers have taken the commercially available tea and are likely to maintain good health. The consecutive ingestion of Guava Leaf Tea with every meal is expected to benefit pre-diabetic and diabetic patients as an alimentotherapy in both developed and developing countries.

## Abbreviations

DM: Diabetes mellitus; T2DM: type 2 diabetes mellitus; T1DM: type 1 diabetes mellitus; FOSHU: foods for specified health uses; GvEx: aqueous guava leaf extract; GvPP: guava leaf polyphenol; alpha-GI: alpha-glucosidase inhibitor; AUC: area under the curve; FBG: fasting blood glucose; BMI: body mass index; HbA_1c_: hemoglobin A_1c_; HOMA-IR: homeostasis model assessment for insulin resistance; T-CHO: total cholesterol; TG: triglyceride; NEFA: nonesterified fatty acid; LDL-CHO: LDL-cholesterol; CYP: cytochrome P450.

## Competing interests

YD hold patents covering the uses of Guava Leaf Tea, the agent for treating renal disorders and elevating adiponectin level.

## Authors' contributions

YD drafted the manuscript, and KM restructured, revised and completed the writing of the manuscript. Both authors read and approved the final manuscript.
